# Methodus Medendi, or the Description and Treatment of the Principal Diseases Incident to the Human Frame

**Published:** 1843-07-01

**Authors:** 


### Methodus Medendi, or the Description and Treatment of the Principal Diseases, incident to the Human Frame. By *Henry M'Cormac*, M.D. Consulting Physician of the Belfast Hospital, and Professor of Medicine in the Royal Belfast Institution. London: Longman and Co. 8vo. pp. 574, 1842.

That Dr. *M'Cormac* is an accomplished medical scholar is abundantly apparent from the character of the present as well as of his former writings. He seems to have made himself acquainted not only with all the current literature of the present age, but also with most of the works of the olden time, from that of the great Father of Medical Science down to the close of last century. How far this elaborate scholarship has subserved his fitness to become an author of a systematic work on practical medicine, we will not venture to determine; but this one thing we may say with confidence that, had he given us less of the opinions of others, and more of the results of his own clinical experience, we fully believe that this present volume would have been much more acceptable to the public, and decidedly more useful to the practising physician. When we first read the title of the work—Methodus Medendi—we had formed to ourselves an idea of a very different work from the present, and had expected to find rather a somewhat original exposition of the general principles of medicine, and more especially of its therapeutic department, than a minute description of its nosological catalogue. It may have been, therefore, partly from this circumstance that the perusal of it has disappointed us a good deal, alike in respect of the subjects that are brought under review, and the manner in which they have been discussed. We had supposed that it might have been similar in its general character to Dr. *Alison's* Outlines, which we introduced to our readers in our last Number, and to the admirable volume which Dr. *Billing* published some years ago. But, instead of this, we have a solid closely-printed volume of nearly 600 pages, commencing with the description of Fever, and—after a history of almost all the ills to which flesh is heir, not even excepting toothache and anaphrodisia—closing with a long account of Mania.

The author has grouped all diseases in the following eight classes:—1. Fevers; 2. Diseases of the respiratory organs; 3. Diseases of the circulatory organs; 4. Diseases of the digestive organs; 5. Diseases of the urinary and genital organs; 6. Diseases of the absorbent system; 7. Diseases of the organs of relation; and 8. Diseases of the nervous system. No classification, we are well aware, will meet all difficulties, or can possibly be exempt from many defects; but we much question whether any arrangement could be more objectionable than the one adopted by our author.

Let us take a few examples:—Cholera is made a fever, while dysentery is a disease of the digestive organs; Scurvy is a disease of the circulatory organs; Hydrophobia is a disease of the absorbent system,—why more so than syphilis? and Anasarca is a disease of the organs of relation! Hydrothorax and ascites do not appear in the catalogue at all; the one being incidentally introduced under the head of pleuritis, and the latter under that of what the author calls sero-enteritis. Then we have Chorea described under the head of epilepsy; and Dyspepsia as if it were a mere form of gastritis.

The 7th class is perhaps the most objectionable of all; as it seems to us to comprehend and groupe together the most dissimilar diseases, while it excludes others which certainly belong to "the organs of relation." Have not erysipelas and urticaria, we may ask, quite as good a title to be ranked among the cutaneous diseases, as eczema or strophulus? and surely that must be a very arbitrary nosological arrangement that places dropsy of the cellular tissue between vermin in the skin on the one hand, and rheumatism and gout on the other. In some instances the classification appears to be not only unwise, but positively erroneous, and calculated to mislead the inexperienced. Not a few cases—perhaps we should rather say, not a few forms—of puerperal fever ought unquestionably to be placed among the Pyrexiæ; and yet all are alike grouped together under one common appellation of metritis or inflammation of the uterus—a term expressive of a most erroneous and hurtful pathological doctrine, if intended to be of universal application.

It appears to us that Dr. *M'Cormac* has erred, in more than one part of his work, by attempting to assimilate and simplify too much various diseases and classes of disease; from his dislike, we should imagine, for the over-minute divisions of many of his predecessors. But then, be it remembered, there is as great an evil in a forced condensation, as in a wearisome amplification, of any subject. Nowhere is our author's mistake in this respect more conspicuous than in the very first chapter of his work; that which treats of Continued Fever. Under the term 'Fever,' he comprehends and describes together the history, symptoms, pathology, and treatment of all the different forms of synochus and typhus—mild, serious, and malignant. The confusion of the narrative, it may be well imagined, is extreme; indeed it is almost impossible for the reader to form any distinct image or picture, if we may so speak, in his mind of what the many-headed Protean monster, that the author is attempting to portray, really is; for its features are ever varying, and withal are so numerous as utterly to defy all attempts at prehension. But let us see what Dr. *M'Cormac* says himself, in order that we may do no manner of injustice to his sentiments. At page 7, it is written :—

"Our knowledge of the pathology of fever is unhappily limited. After describing the complaint, its varieties, complications, and making a few inferences beyond, little remains to be said. We know nothing of the *indoles* of the complaint, or its proximate cause—why it lasts a given period, and then declines. Fever is a disease of the whole system; it affects the solids and the fluids, the organs of digestion, circulation, respiration, and relation; it affects the body, and it affects the mind. It is a disorder with peculiar characters, often propagating itself by a poison, which, not unlike a leaven, sets up a new series of actions in the system. In other respects, individuals are variously affected: in some there is inflammation, in others none; some get better and recover, while others grow worse and die. The intestinal follicles are inflamed in one person, the lungs in another, while the brain is affected in a third. A few perish in whom there is no discoverable lesion, while others gain health and strength in cases where organic derangements have been numerous and severe. As fever pursues its course, there is progressive deterioration of structure and function; yet, for all this, when the disease is at its height, when destruction seems to impend, it quickly or slowly takes a turn, and all does well." 8.

And again, a little further on, we find our author saying,

"Those writers, who, like Pinel and the Franks, divide fever into numerous varieties, complicate and embarrass the treatment. According to such, we no longer have a given malady, with certain symptoms and complications to deal with, but a number of distinct affections, bearing such epithets as mucous, nervous, putrid, adynamic, ataxic, and typhoid. I would not advocate a simplicity that nature did not sanction; but, assuredly, fever does not present varieties more numerous than most other diseases. From whatever cause the malady may arise, the treatment is mainly alike." 17.

We need scarcely comment upon these passages. With a good deal of shrewd and just remark in them, there are one or two very glaring errors that cannot fail to attract the notice of every reader—witness the concluding paragraph. We had marked several other passages for notice; but perhaps this is unnecessary; as the whole chapter requires a very thorough revision. That Dr. *M'Cormac* is fully competent to write ably on any medical subject to which he directs his attention, we are quite confident; but, to do it justice, he must consent to no inconsiderable labour in arranging under clear and distinctly marked heads the mass of materials which he has collected together.

He is evidently a man of highly cultivated mind, and of liberal and most enlightened views; and withal, we should presume, a sound practical physician. We observe too, that he is a public teacher of his profession. Is it the case that the present volume is made up of the notes or short memoranda used by him as aids to memory in his prelections?—This idea certainly suggested itself to our mind more than once in reading the present volume.

Most systematic works on the practice of medicine open with some prefatory observations on the animal economy; the composition and contexture of its different parts; its active powers in health; the changes to which these are subject from a variety of disturbing influences; its reparative energies, whether spontaneous or promoted by art; and so forth. Dr. *M'Cormac* would not have done amiss, had he followed the good old path of his predecessors, even at the risk of censure as a mere imitator.

If, instead of plunging all at once *in medias res, i. e.* the description of particular diseases, he had given a brief but lucid exposition, in an introductory chapter, of the leading and characteristic features of the various classes of his nosological catalogue, and of the particular states of the system with which these are generally associated—and a right knowledge of this, by-the-bye, is essential to any rational system of therapeutic instruction—he would have saved himself much unnecessary repetition of detail, and his readers not a little embarrassment in following him. In the review of Dr. *Alison's* work, in our last Number, we pointed out the due importance which this excellent author has attached to the morbid changes of the blood as intimately connected with the ætiology of many diseases; and we *then*—as indeed on many former occasions—expressed our own convictions that no truly scientific exposition of the principles and practice of medicine can be given in the present day, without considerable attention being paid to a Humoral system of pathological doctrine. The recent researches of *Liebig* on the one hand, and of M. *Andral* on the other, have powerfully contributed to direct the enquiries of medical men into this channel. Dr. *M'Cormac* has, indeed, alluded to the subject; but certainly not in the manner, or to the extent, that it deserves. Thus almost all that we are told of the altered condition of the blood in fevers is that "the mass of solids and fluids is diminished; the vitiated blood is imperfectly arterialised, and, from being copious, rich and fluid, becomes poor, serous and dark-coloured;" and again; "the blood, in the course of fever, undergoes certain changes to which the terms watery, dissolved, and putrid have been applied. A living fluid, like the blood, cannot become putrid; but alterations do occur, which exercise much influence on the disease."

We shall now make a few selections upon different topics, in order that our readers may judge for themselves of the general scope and character of the contents of the present work.

### *Results of Treatment in Fever.*

"Were the number of writers and of remedies a criterion, fever should be most amenable; as it is, however, nature is the principal physician. And it is to be feared that the curative measures, hitherto instituted, have not materially lessened the aggregate mortality. The frequency of epidemies removes the disease, in a measure, beyond the pale of treatment, and medical men themselves are carried off in numbers. During the former, however, the aggregate mortality, is great, but the comparative mortality is notably less. Well-concerted measures would lessen, if not do away with these dreadful visitations, but the defective structure of society would go far to neutralize such at present. However this may be, the practitioner is enabled to study individual cases with their complications, and by judiciously adapting the resources of his art, does much as circumstances permit, towards their mitigation and removal. Sydenham has been slavishly followed in his dictum, as to the variety observable in the character, and, therefore, in the treatment of febrile epidemies. The constitution of the air, I suspect, has less to do than the constitution of the patient, and the condition of the community wherein the disease prevails. The same variety is observable during epidemies, as at other times; abdominal affections prevail in Autumn, and pulmonary ones in Spring. In other respects, typhus is commonly less frequent in Summer than Winter." 16.

Dr. *M'Cormac* is, we think, quite in error, when he under-rates the importance of what the English Hippocrates called the constitution of the air or season, in reference to the mildness or gravity of different epidemics. The experience of every year amply confirms the perfect truth and practical value of the old physician's views on this subject.

### *Influence of the Mind on Agues.*

"Intermittents, it appears, are sometimes removed by mental impressions. The prince of Saxe-Wiemar experienced quotidian at mid-day, which resisted every mode of treatment. Hufeland, his physician, put the clock two hours forward, and the overjoyed patient, believing himself cured, became so in reality. Charms and secret nostrums have proved not altogether impotent: most have heard of Judge Holt and his ball. The fear of a tempest has checked an accession of ague; and a friend of mine recovered instantly on learning that his ship was on fire. Stokes mentions, that the fits often failed to ensue, when patients were directed to be bled in their hearing. Pliny tells of a captain whom an engagement released; and Joseph Frank, of a soldier who was frightened into the disorder by one battle, and out of it by another. An attack has taken place when the patient anticipated the customary hour; thus, Riverius relates the case of a man, who having gone out to ride, heard a steeple chime an hour more than it really was, whereupon, back came his ague on the spot." 34.

The energy of a resolute mind will certainly often go a great way in checking the return of intermittent attacks; and, on the other hand, fear, despondency, and worrying anxiety, will inevitably cause them to take a stronger hold and a deeper root in the constitution. Colonel *Napier,* or some other historian of the Peninsular War, mentions an anecdote of the *Duke of Wellington,* that bears upon this point. It is well known that the great chief himself never left the Peninsula—from the time of the British troops landing on the coast of Portugal, in 1809, till they triumphantly planted their standards on the French soil in 1814—although there was scarcely an officer of high rank in his army who had not been obliged by sickness to obtain furlough of leave. The *Duke* had repeated threatenings of ague attacks; and how did he treat them? Just before the expected fit, he would take a draught of laudanum and æther, mount his horse, and ride at full speed for a couple of hours, until he was in a state of free perspiration.

Dr. *Billing,* if we remember aright, has very happily pointed out, in his clever work on the Principles of Medicine, the points of analogy between ague and neuralgia.* Some of these will naturally occur to every medical reader; but perhaps none is more convincing than the marked influence of the mind on the progress of both diseases.

### *Treatment of Hooping-cough.*

The account given by the author is certainly very unsatisfactory. As a matter of course, he, like every other physician, inculcates the necessity of antiphlogistic measures in the early stage, when there is usually some bronchitic or pneumonic tendency existing. The treatment at this period of the disease is abundantly obvious and simple. It is when the fits of coughing remain frequent and severe, all the inflammatory symptoms having quite passed away, that the chief difficulty is experienced.

What is best to be done, then? According to our author, little good is to be expected from medicine. Change of air, attention to the state of the bowels and skin, and an occasional emetic, seem to him to be the chief means of relief. He has tried, he tells us, prussic acid, belladonna, and other antispasmodics; but has never found them to have any decided effect in allaying the attacks, or in shortening the duration of the disease. He does not even allude to the use of the alkalies, a very safe and often a most important class of remedies;† nor to that of tonics, such as quinine, the salts of iron, zinc, &c. although these are often of

---

* Some remarks on this subject from the pen of M. *Piorry,* one of the physicians of La Pitié Hospital in Paris, will be found in the Foreign Periscope of the present number.

† In our own practice we are partial to a mixture of nitrate of potash, or of the sub-borate of soda, with hydrocyanic acid, compound camphor tincture, unquestionable advantage, especially when combined with conium, or some other of the milder sedatives.

*Dyspepsia.*

We select the following passage as a very fair, and withal a favourable, specimen of our author's style and mode of treating his theme—short, pithy, and expressive. The general tone of the remarks reminds us much of the introductory chapter to the well-known "Change of Air," by the Senior Editor of this Journal—a book, by-the-bye, which we should very cordially recommend to most dyspeptic patients.

"It is plain, from the importance attached to purgatives, that the majority of writers have looked on them as the essential; exercise in the saddle, however, or on foot, morning frictions and the shower-bath, with a plain restricted regimen, will do more good than all the purgatives in the world, useful though they be, without these means. Walking, progressively increasing the amount, is one of the best restoratives imaginable; it is even superior to horse exercise, so much and so justly lauded by our English Hippocrates. Muffling, in the shape of flannel next the skin, is better than a great coat; and with a stout frock, a stick, and, if possible, a cheerful companion, were it only a dog, let the patient sally forth—if over a rugged unequal surface, so much the better. Languor will at first ensue, but, as this goes off, an exhilarating glow pervades the frame, ending in cheerfulness, craving appetite, and, if properly persevered in, complete renovation of all the excretions. I sometimes advise a patient to walk one day and ride the next; indeed, passive exercise is often useful. It may happen that entire change of scene and occupation is desirable; the greater, the more effectual. In 1821, a student harassed with dissections at La Pitié, clinique at the Hôtel Dieu, and midnight study, lost all desire for food; his colour grew bad, his spirits languished, and the bowels became obstinately constipated. In this state, he drove off, *summa diligentia*, to Brussels, where he wandered about the environs and ate spring fruit without reserve, but of other medicine none; in a fortnight's time, no trace of indisposition remained. The dyspeptic labourer only requires better food, the dyspeptic artisan better food and air. If the poor tiller of the soil could but subsist on the fat of the earth, and the rich sybarite be reduced to hard fare and assiduous toil, it would equally benefit both. The literary dyspeptic should live more *sub dio—melior est ambulatio sub divo, quam in porticu,* quoth Celsus. A man's mind was not intended to be a mere receptacle for book lore—a literary sieve; the body must be cultivated likewise. Let study be followed in the morning, at night never; and let four or six hours daily be spent in the open air. The student's motto should be σπευδε βραδεως, *festina lente*—the *ohne Hast aber ohne Rast* of the illustrious Göethe; thus, as life wore away, his mental would increase without prejudice to his corporeal powers." 247.

*Remarks on Sterility.*

We were much struck with an observation made in the late official report, by the Poor-law Commissioners, on the Sanitary Condition of the Labouring Population in Great Britain—of which a pretty full analysis is given in our last Number—that, so far from the great mortality in large

---

ipecacuan wine, and dill water. In a great number of cases, it has proved of singular efficacy. If the child be very weakly, the decoction of bark may be the vehicle, or small doses of quinine may be given at the same time.

towns proving a check to the increase of population, it is found that the proportion of births to the number of inhabitants is actually higher there than in rural districts, where the comparative number of deaths is much smaller. "In Manchester," we are told, "where one twenty-eighth of the whole population is annually swept away, the births registered amount to one in 26 of the population; while, in the county of Rutland, where the proportion of deaths is one in 52 of the population, the proportion of births is only one to 33."

Mr. Chadwick subsequently proves, by the results of last year's registration, that "there has been an increase of the population from births alone in those parts of the country where the proportionate mortality is greatest."

The following remarks, by Dr. *M'Cormac*, will be read with interest in connexion with these rather unlooked-for results.

"It is remarkable," he says, "that habitual excess in eating and drinking proves adverse to fertility; and such, I take it, is the principal source of the sterility of the rich; this has even been observed in the inferior animals. Habitual plethora seems to indispose to fruitfulness; while poor people, with a bare sufficiency, if even that, have houses swarming with offspring. The prolific tendencies of the Irish are well-known: barren marriages are rare among them. Fish-eaters, icthiophagi, who have to work hard, and live without excess, are proverbially fruitful. Hence it is that the reproductive tendencies of a people—a problem vainly attempted by Malthus and others, is in the inverse ratio of their comforts, and not merely of their numbers, as conjectured by Sadler. Hence, also, the poorer and more wretched a population, within certain limits, the more prone to increase. In other respects, the poor follow, but do not precede the promptings of nature. The rapidity with which the void left by famine, war, and epidemies, fills up, has been frequent matter of observation, and is readily enough explained by the increased facilities of subsistence and consequent inducements to marriage among survivors. In two or three instances, by enjoining separate apartments for a time, simple sparing regimen, the shower-bath, and exercise in the open air, I have reason to believe that the wishes of parents, previously childless, were successfully promoted." 350.

With another extract, and it is a valuable one, we shall close our notice of the present volume.

*Connexion between the Functions of the Skin and the Urinary Organs.*

"The emunctory for carbon in the human body is the lungs—for azote, merged in uric acid, the kidneys—occasionally, as in gouty subjects, the joints. The occasional conjunction of gout, uric acid gravel, and stone, is notorious. How far the functions of the kidneys and skin are convertible in this matter, is difficult to say; but, certain it is, that those who maintain due cutaneous transpiration, are much less liable to lithic acid deposits. In connexion with this subject, I may advert to an interesting observation of Wilson Philip, that acescent food, ordinarily productive of a lithic acid deposit, had no such influence when due action of the skin was maintained by diaphoretics. It is obvious, that excess of azote is consumed in muscular nutrition, a process greatly enhanced by exercise. Sailors, soldiers, labourers, huntsmen, pedestrians—the Esquimaux live on it exclusively, consume azotized food without sustaining inconvenience, but the case is far otherwise with the indolent and replete. Many such, as I have observed, habitually void urine surcharged with uric acid. Indeed, I know those who never discharge clear urine except when they grow ill, and when they become unable to continue their wonted excess. Brodie mentions a gentleman, who, though he lived freely, sweated himself by morning exercise; this interrupted, his urine began to display red gravel, his skin a scaly eruption. Majendie tells us of a merchant of the Hanse Towns who discharged uric acid gravel while his business was prosperous, becoming clear when it declined; and conversely, returned to its old condition when his affairs rallied. But the majority, if they will take the trouble, may observe these alternations in themselves or others." 322.

When we next meet Dr. *M'Cormac* as an author, we trust that we may have it in our power to bestow more unqualified praise on his work. His is not a character to submit to the tedious drudgery required in compiling a systematic Treatise on Medicine; it is too brisk, off-hand and impatient. His talent, we should say, is for clever animated monographs.

